# Crohn’s Disease Prediction Using Sequence Based Machine Learning Analysis of Human Microbiome

**DOI:** 10.3390/diagnostics13172835

**Published:** 2023-09-01

**Authors:** Metehan Unal, Erkan Bostanci, Ceren Ozkul, Koray Acici, Tunc Asuroglu, Mehmet Serdar Guzel

**Affiliations:** 1 Department of Computer Engineering, Ankara University, 06830 Ankara, Turkey; mthnunal@ankara.edu.tr (M.U.);; 2 Department of Pharmaceutical Microbiology, Faculty of Pharmacy, Hacettepe University, 06230 Ankara, Turkey; 3 Department of Artificial Intelligence and Data Engineering, Ankara University, 06830 Ankara, Turkey; 4 Faculty of Medicine and Health Technology, Tampere University, 33720 Tampere, Finland

**Keywords:** microbiota, Machine Learning, bowel disease, bioinformatics

## Abstract

Human microbiota refers to the trillions of microorganisms that inhabit our bodies and have been discovered to have a substantial impact on human health and disease. By sampling the microbiota, it is possible to generate massive quantities of data for analysis using Machine Learning algorithms. In this study, we employed several modern Machine Learning techniques to predict Inflammatory Bowel Disease using raw sequence data. The dataset was obtained from NCBI preprocessed graph representations and converted into a structured form. Seven well-known Machine Learning frameworks, including Random Forest, Support Vector Machines, Extreme Gradient Boosting, Light Gradient Boosting Machine, Gaussian Naïve Bayes, Logistic Regression, and k-Nearest Neighbor, were used. Grid Search was employed for hyperparameter optimization. The performance of the Machine Learning models was evaluated using various metrics such as accuracy, precision, fscore, kappa, and area under the receiver operating characteristic curve. Additionally, Mc Nemar’s test was conducted to assess the statistical significance of the experiment. The data was constructed using k-mer lengths of 3, 4 and 5. The Light Gradient Boosting Machine model overperformed over other models with 67.24%, 74.63% and 76.47% accuracy for k-mer lengths of 3, 4 and 5, respectively. The LightGBM model also demonstrated the best performance in each metric. The study showed promising results predicting disease from raw sequence data. Finally, Mc Nemar’s test results found statistically significant differences between different Machine Learning approaches.

## 1. Introduction

Machine Learning (ML) is a subfield of Artificial Intelligence (AI) that focuses on developing algorithms and statistical models that enable computer systems to learn and improve from experience, without being explicitly programmed. ML algorithms can be trained on large datasets to identify patterns and relationships that can be used to make predictions or decisions about previously unseen data.

In the context of disease detection, ML can be used to analyze medical data, such as medical images, electronic health records, or genetic data, to identify early signs of disease or predict the likelihood of developing a particular disease. ML algorithms can also be used to develop diagnostic tools that can accurately detect diseases in patients.

In the last 30 years, genetic sequences of numerous living organisms have been discovered and uploaded to online databases. Therefore, it is now easier to analyze and evaluate these organisms based on their sequences.

The human microbiota, which consists of trillions of microorganisms that live in our bodies, has been found to play an important role in human health and disease. Metagenomics and microbiome sciences have undergone a revolution thanks to high-throughput sequencing. Moreover, analyzing microbiome data using ML algorithms can provide new insights into the role of microbiomes in health and disease.

In this study, modern ML models were trained with genetic sequences obtained from the human microbiota to predict whether individuals are healthy or not. For this purpose, the sequence dataset of Gevers et al.’s [1] study which includes 16S rRNA amplicon sequence of individuals with Inflammatory Bowel Disease (IBD) was downloaded from the National Center for Biotechnology Information (NCBI) [2] database.

Firstly, De Bruijn graph representations were generated using the sequence dataset. Secondly, these representations were converted to a structured form which is one of the novel parts of this study. Afterwards, this structured data was used to train 7 widely-known ML frameworks namely, Random Forest, Support Vector Machines, Extreme Gradient Boosting, Light Gradient Boosting Machine, Gaussian Naïve Bayes, Logistic Regression and k-Nearest Neighbor. The hyperparameters of these models were optimized using the Grid Search algorithm. The results were evaluated using accuracy, precision, fscore, kappa and area under the receiver operating characteristic curve (AUC) metrics for each model. Finally, Mc Nemar’s test was employed to assess the statistical significance of the experiment.

In this study, we designed a novel way to represent the sequences in graph based structured form and used this structured data to train state-of-the-art ML models like Light GBM, XGBoost etc. We also evaluated the results with popular metrics and tested the statistical significance with Mc Nemar’s test. This study also demonstrated that raw sequences can be used without extracting OTUs (Operational Taxonomic Units) to classify diseases.

The rest of the paper is constructed as follows. Section 2 demonstrates the literature of ML algorithms and how they are used in sequence-based analysis of diseases. Section 3 presents the material and method of the study which includes a detailed examination of the dataset, the data preprocessing phase and ML models and optimization method used in the study. The prediction results of the ML models are presented and discussed in Section 4. In Section 5, the study is concluded and future work is presented.

## 2. Literature Review

Microbiota refers to the community of numerous microorganisms, including viruses, bacteria, and fungi, that use the human body as a host [3]. Because it affects metabolism and immunity, microbiota is crucial for human development [4]. To provide and maintain homeostasis within the human body, humans and their microbial community have evolved together to be in constant communication and partnership [5]. The human microbiota is in perpetual competition with pathogens to maintain a colonization resistance and help regulate the immune response [6]. However, the issue of what ensures the longevity of a healthy microbiota remains controversial.

Recently, many studies suggest that the risk of various disease like cancer [7], diabetes [8], obesity [9], and autism [10] may be caused by imbalances in the gut microbiota. This imbalance in the gut microbiota is defined as dysbiosis [11]. As the human microbiota play an essential role in disease and health, they can be used as biomarkers and provide insight into the pathology of certain diseases [12]. Therefore, it is a significant problem to predict diseases that may occur in the host with the data obtained from the microbiota [13]. Machine Learning algorithms have proven to be effective in solving such prediction problems [14].

Inflammatory Bowel Disease (IBD) is a group of disorders that are characterized by chronic inflammation of the gut. Ulcerative Colitis (UC) and Crohn’s Disease (CD) are the two main types of IBD. The imbalance in the gut microbiota may lead host-mediated inflammatory responses and promote IBD development [15]. As differential diagnosis of IBD is challenging, ML models using microbiome data may be a promising approach for the diagnosis.

It is also worth mentioning genetic sequences and next-generation sequencing. With the development of next-generation sequencing technologies and the completion of the Human Microbiome Project, a lot of new information has been gained regarding the microbiota and its functional properties [16].

Due to little evolutionary change, 16s ribosomal RNA (rRNA) has become the most important DNA region for identifying microorganisms [17]. The 16S rRNA gene is comprised of nine hypervariable regions (V1–V9) [18]. Targeting one to two hypervariable regions for next-generation sequencing is a widely used approach in microbial community profiling.

The 16S rRNA gene sequences from microbiome studies are usually publicly available in online databases such as the NCBI Sequence Database [19]. The NCBI database contains millions of short sequences for many species in different formats [20].

In recent years, ML has been increasingly used to analyze amplicon-based and whole genome shotgun sequencing microbiome data [21]. The first example of how sequences from microbiota are used in ML includes microbial identification [22]. ML algorithms can be trained on microbial DNA sequences to accurately identify the bacterial species present in a given sample. This can help to understand the diversity and composition of bacterial communities, and to identify potentially pathogenic bacteria [14].

Another example is treatment response which includes ML algorithms to analyze microbiota data to predict how patients will respond to certain treatments, such as antibiotics or probiotics [23]. This can help to personalize treatment plans for patients and to optimize therapeutic outcomes [24].

One of the most important usages of ML algorithm on sequence data is disease prediction [25]. ML algorithms can be trained on microbiome data to predict the likelihood of developing certain diseases or conditions, such as inflammatory bowel disease [25], diabetes [26], or cancer [27]. These predictive models can be used to identify patients who are at high risk for developing these diseases and to provide targeted preventive interventions [28].

Literature presents different usage of 16s rRNA sequences with ML algorithms. Chaudhary et al. [29] developed a tool using the RF model for taxonomic classification of 16S rRNA sequence. The tool achieved over 99% accuracy for taxonomic prediction on both the genus and phylum models.

Solis-Reyes et al. [30] developed an open-source k-mer based ML tool for subtyping of HIV-1 sequences. The tool uses k-mer frequencies of the sequence reads without alignment with Support Vector Machine, Multilayer Perceptron and Logistic Regression models to determine the subtypes of HIV-1.

Nakano et al. [31] used samples obtained from oral mucosa to predict oral malodour employing Deep Learning (DL) and SVM. These samples include Operational Taxonomic Units (OTUs) of 16s rRNA sequences rather than raw sequences. The study uses samples from only 90 individuals (45 of them marked oral malodour) which can be considered as not enough data for DL models learn. 

One of the first examples of using raw sequences rather than OTUs is Asgari et al.’s [32] study. In this study, 16s rRNA sequences and OTUs were used to train ML models and predict different diseases. The study proposed an alignment-free method for genetic sequences to use in ML models. This k-mer based method utilized shallow sub-samples to generate features which were later applied to train a Random Forest (RF) model. This study also used Gevers et al.’s [1] Crohn’s Disease dataset and obtained 75% and 76% precision for k-mer lengths of 5 and 6, respectively.

Topçuoğlu et al. [33] designed a framework including RF, SVM, LR and XGBoost to predict the presence of neoplasias. The study employed area under the receiver operating characteristic curve (AUC) as the evaluation metric and RF demonstrated the best performance regarding the 0.695 AUC score. The tool developed here also provided a pipeline to train, test and interpret the results.

Although literature presents many applications of ML based on OTUs, the studies that use raw sequences based on k-mer representations are very rare.

## 3. Materials and Methods

In this study, we designed an ML system to detect IBD using genetic sequence data. Firstly, the sequence dataset was downloaded from an open access NCBI database. The fastq files were cleaned to obtain raw sequence files. These files contain thousands of raw genetic sequences. In the third stage, these sequence reads were converted to the k-mer based structured graph representation form. In the next stage, this structured dataset was divided into train and test subsets. The Grid Search algorithm was used to optimize the hyperparameters of the ML models. Then, ML models with optimized hyperparameters were trained with a train subset of the dataset. Finally, the test subset was used to measure the performance of the ML models.

**Figure 1:**
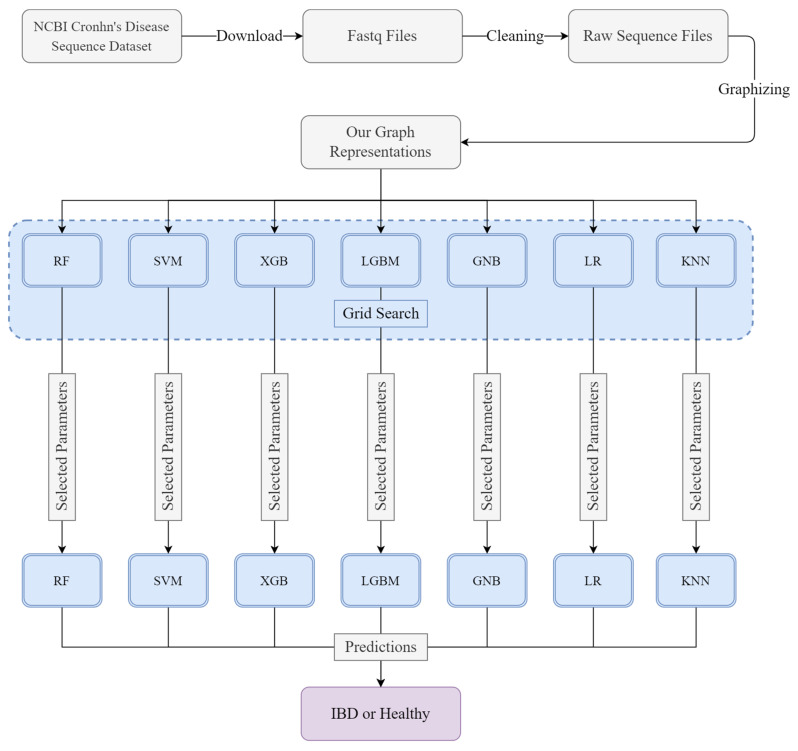
General scheme of the study.

This section contains detailed examination of the dataset, preprocessing stage and employed ML models. The general framework of the study can be seen in Figure 1.

### 3.1. Sequence Dataset

The dataset used in this paper is the dataset of Gevers et al.’s [1] study which is obtained from the NCBI database (Bioproject PRJEB13679 [1]). This dataset consists of V4 hypervariable region sequencing data from a total of 1359 samples. Each sample is stored in this database in sra or fastq formats. The fastq format, which is easier to read and process, is preferred.

**Figure 2:**
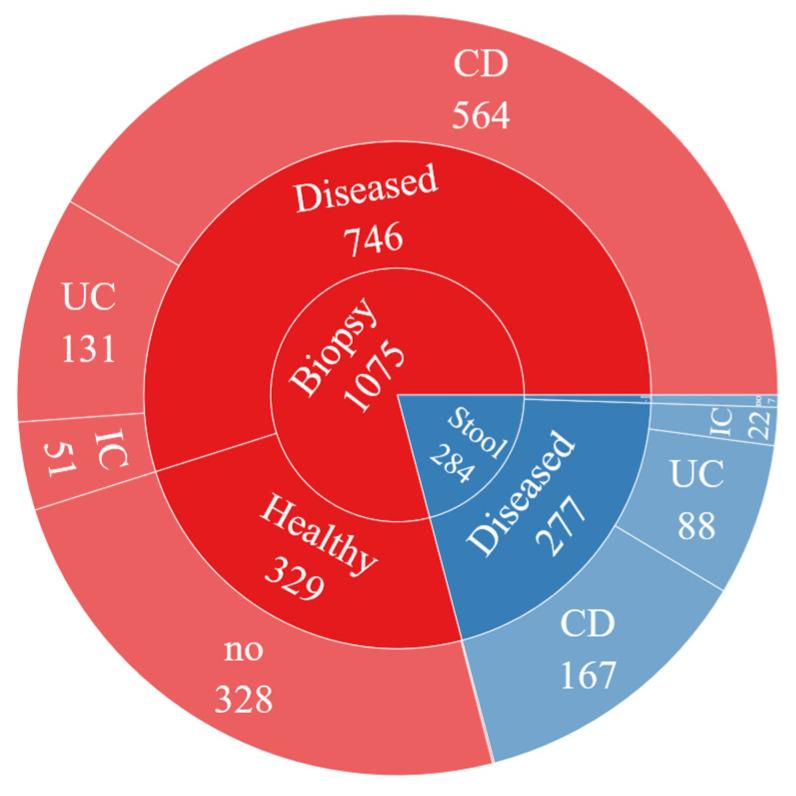
Distribution of the dataset over the method of obtaining.

The sequences in the dataset were obtained from biopsy and stool samples. The distribution can be seen in Figure 2. Accordingly, 1075 (79%) of 1359 samples in the dataset were obtained by biopsy and 284 (21%) were obtained from stool samples. IBD was diagnosed in 746 (69%) of the people from whom biopsy samples were taken, while the remaining 329 (31%) did not have this disease. In addition, 277 (97.5%) of the participants whose stool samples were collected were diagnosed with IBD, while the number of those who did not have the disease was limited to 7 (2.5%).

**Figure 3:**
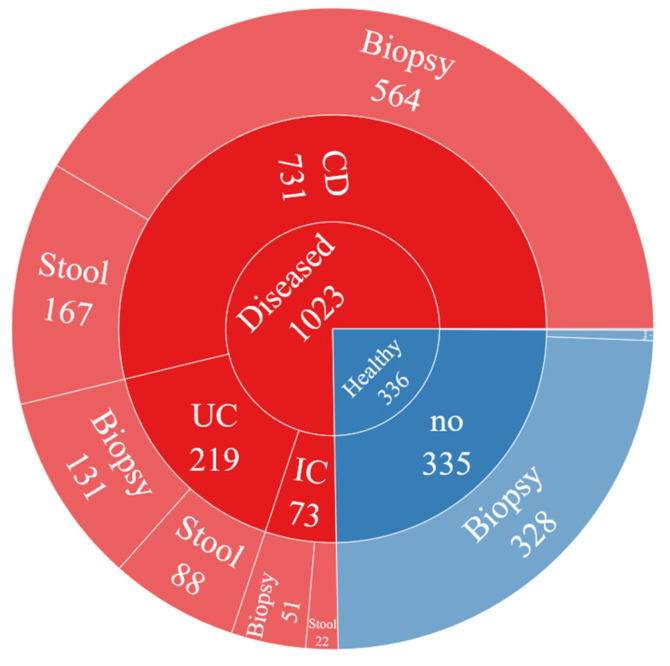
Distribution of the dataset considering disease status.

The distribution of this dataset according to the diagnosis status can be seen in Figure 3. Accordingly, 1023 (75%) out of 1359 people were diagnosed with IBD, while 336 (25%) were healthy. Here, CD, UC, and IC are various forms of IBD. It should be mentioned here that two of the 16S rRNA sequence files were not used in this study because they contain very few reads.

**Figure 4:**
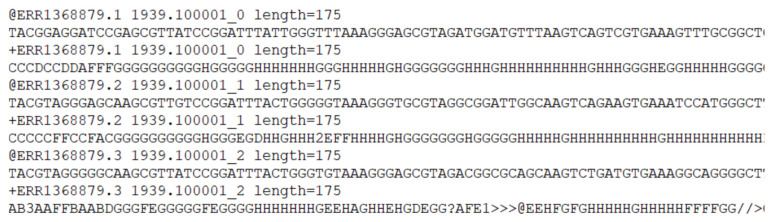
Contents of sample fastq file.

As mentioned earlier, each file in the dataset is in fastq format. A fastq file contains 4 lines of information for each sequence. The first line is the sequence identifier and starts with the “@” symbol, the second line contains the raw sequence data, the third line starts with the “+” symbol following same sequence identifier, and the quality scores is located in the last line. The contents of a sample fastq file can be seen in Figure 4. Each of the 4 lines contains information of one sequence read.

### 3.2. Data Preprocessing

**Figure 5:**
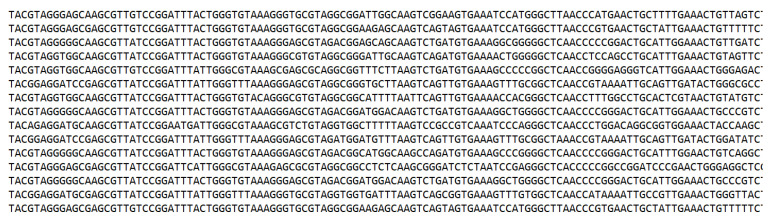
Contents of a file after removing extra information.

As can be seen in Figure 4, a fastq file contains not only raw sequence but also extra information about the read. This study only uses the raw sequences, therefore the extra information of each sequence in each file was removed and the file converted to a standard text file. The final state of the raw sequence file can be seen in Figure 5.

In the second stage of the data processing, the raw data sequences in each file were converted to the graph representations. This stage includes 2 sub-stages which convert sequences to De Bruijn graphs by selecting k-mer lengths and converting these graphs to our graph representation.

Here, it is useful to mention De Bruijn graphs. In a De Bruijn graph, the nodes represented by subsequences of length k, called k-mers, are taken from the original sequence [34]. The edges in the graph connect two nodes if there is a k-1 overlap between the corresponding k-mers.

To generate De Bruijn graph representations, k values 3, 4, and 5 have been selected. Each file is converted to a single graph which is represented by source and destination nodes with edge weight. The weight parameter corresponds to the number of times each edge exists in a file.

For the next step, the separate graph representation is combined to generate a single file for each k value. These new files contain four features namely, graph id, source id, destination id, and weight. The first feature graph id corresponds to the id of the sequence file of an individual on the dataset. The source id and destination id represent the k-mer nodes in related edge.

**Figure 6:**
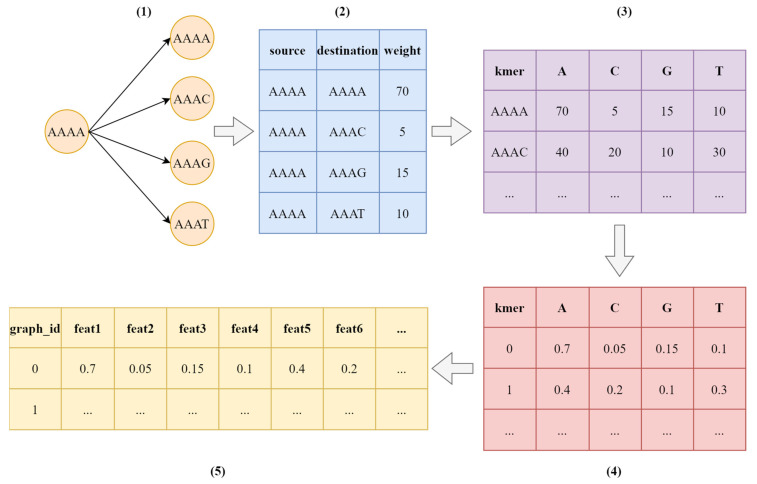
One-line representation process of a sequence graph (k = 4 for k-mer).

ML and DL models require structured files to be trained. For this purpose, the dataset has to be converted to standardized structure. We designed a novel way to represent sequence files in De Bruijn graphs. Due to the nature of the De Bruijn graph, there is only one character change between its two edges. It is also worth remembering that there are only 4 different characters in a sequence. Hence, there are only 4 different nodes to go after each edge, and the last character added in these nodes can be one of the 4 bases (A, C, G, T). Therefore, a structure with all possible nodes and 4 bases to represent the next node can be used for training ML and DL models. This structure of the dataset can be seen in (3) and (4) of Figure 6.

This structure is also enhanced by flattening the sequence data of each individual and combining all individuals’ data in one file for each k value ((5) of Figure 6).

In the final phase, the dataset was divided into training (80%) and test (20%) sets. The training set will be used to train, and optimization of the ML models and the test set will be used to demonstrate the performance of ML models on previously unseen data.

### 3.3. Machine Learning Analysis

#### 3.3.1. Machine Learning Models

In this study, seven different ML methods were employed namely, Random Forest, XGBoost, LightGBM, Support Vector Machine, Gaussian Naïve Bayes, Logistic Regression, and k Nearest Neighbor.

Random Forest (RF) [35] is a Machine Learning algorithm that belongs to the family of ensemble learning methods, which combine the predictions of multiple individual models to make more accurate predictions. RF creates an ensemble of decision trees, and each decision tree is built using a random subset of the original features and a bootstrapped sample from the training data. This random feature selection and sampling help introduce diversity among the trees in the forest.

XGBoost (Extreme Gradient Boosting) [36] is an advanced Machine Learning algorithm that is based on the gradient boosting framework. The algorithm is known for its exceptional performance and interpretability. XGBoost builds an ensemble model by combining multiple weak learners, which are decision trees in the case of XGBoost. Initially, the ensemble is empty, and it starts by creating the first decision tree. This tree is often a simple shallow tree with a small number of levels. After the first tree is built, the residuals are calculated. These residuals represent the errors made by the current ensemble model. XGBoost is known for its exceptional performance due to its optimization techniques, regularization methods, and ensemble learning approach.

LightGBM [37] is a gradient boosting framework that is specifically designed for efficient and accurate classification tasks. It is known for its high speed and scalability, making it a popular choice for handling large-scale datasets. Like the above algorithms, LightGBM also builds an ensemble model by combining multiple decision trees. However, it uses a different approach called gradient-based one-side sampling to select and train the decision trees in a more efficient manner. LightGBM’s efficiency and scalability make it particularly suitable for handling large datasets with a high number of features.

Support Vector Machine (SVM) [38] is powerful Machine Learning models that aim to find an optimal hyperplane which separates the two classes in the feature space. SVMs are known for their ability to handle high-dimensional data, handle non-linearly separable cases through kernel functions, and have good generalization properties.

Gaussian Naïve Bayes (GNB) [39] is a simple yet effective algorithm that is based on the Bayes’ theorem and assumes that the features follow a Gaussian (normal) distribution. GNB is known for its simplicity, speed, and ability to handle high-dimensional datasets. However, it makes the strong assumption of feature independence, which may not hold in all cases. Despite this limitation, GNB can still perform well in many practical scenarios.

Logistic Regression (LR) [40] is a popular Machine Learning algorithm which models the relationship between the features and the binary outcome using a logistic function. This function allows for the estimation of the probability of belonging to a particular class. Logistic Regression is known for its simplicity and ability to handle both numerical and categorical features. 

k-Nearest Neighbors (kNN) [41] is a simple yet effective Machine Learning algorithm which classifies new data points based on the majority vote of their k nearest neighbors in the feature space. kNN is known for its simplicity, ease of implementation, and ability to handle non-linear decision boundaries. However, it can be computationally expensive, especially when dealing with large datasets, as it requires computing distances for each new instance.

#### 3.3.2. Hyperparameter Optimization

Each of the above-mentioned models has a different number and variety of hyperparameters. In order to produce results with higher accuracy, the best hyperparameters must be found. For this purpose, hyperparameter optimization was performed using the Grid Search algorithm.

Grid Search [42] is a technique used in Machine Learning to systematically search for the best combination of hyperparameters for a given algorithm. Grid Search involves defining a grid of hyperparameter values and then evaluating the performance of the model for each combination of these values. The performance is typically measured using a specific evaluation metric, such as accuracy, precision, recall, or F1-score, depending on the nature of the problem.

## 4. Results and Discussion

In this section, the results of the ML analysis of this study will be presented. Seven ML models are employed, and Grid Search is used to optimize these models. For evaluation purposes, five metrics are used namely, accuracy, precision, fscore, kappa and Area Under the ROC (receiver operating characteristic). All metrics mentioned here are grouped based on the k value of k-mers.

Firstly, the k-mer length is selected as 3 and the dataset has been processed using the above-mentioned methods. When the k-mer length is 3 and 4 available bases (A, C, G, T), it is clear that 64 different k-mers can be found in a sequence file. According to the processing method in Figure 6, the length of the sequence representation of one individual is 256 (64×4). After the preprocessing stage, the dataset contains the data of 1357 individuals with 256 features for a k-mer length of 3. From this it can be easily understood that the number of features for k-mer lengths of 4 and 5 are 1024 and 4096, respectively.

**Figure 7:**
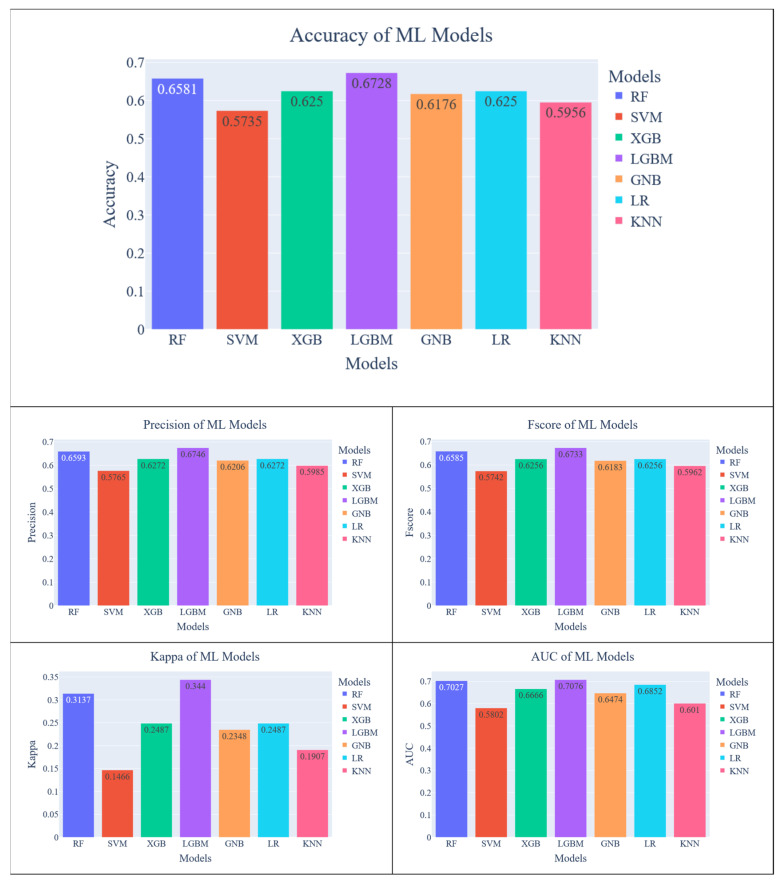
Evaluation results of ML models for k = 3.

The evaluation results of a k-mer length of 3 can be seen in Figure 7. Regarding accuracy, the most successful result was obtained from the LightGBM model with 67%, followed by RF model with 65%. These two models outperformed others in each metric. Also, the AUC score of these two models is quite close. While the difference between the success of the models in accuracy, precision and fscore metrics is small, the difference is more clearly seen in the Kappa metric. On the other hand, the least successful result was obtained from the SVM model with 57% which also shows worst performance regarding other metrics. 

**Figure 8:**
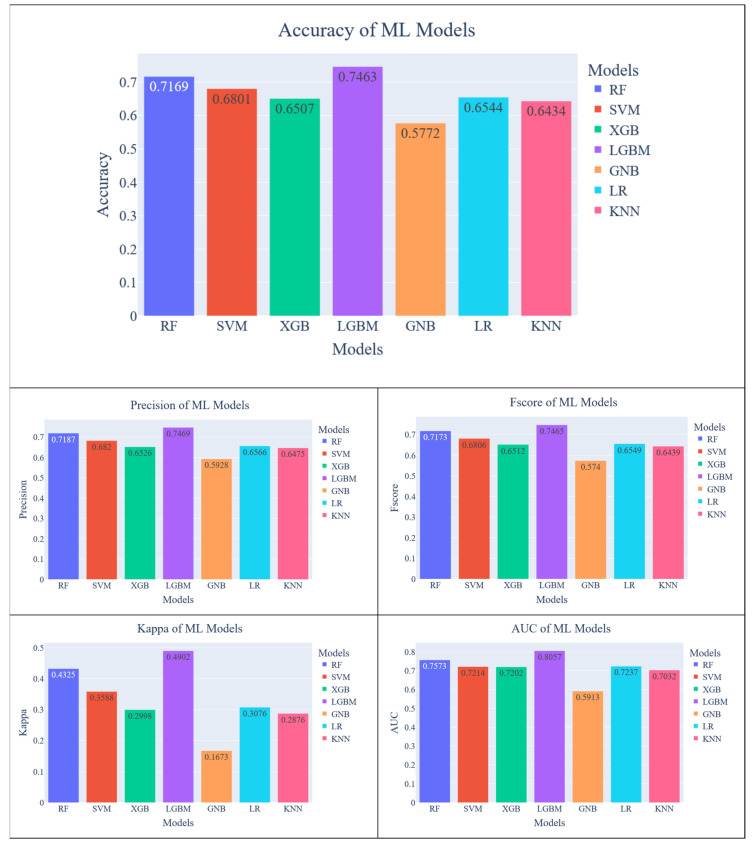
Evaluation results of ML models for k = 4.

In the second phase, the k-mer length set as 4 and the dataset is used to evaluate the models all over again. As can be seen in Figure 8, The LightGBM model achieved 74.63% accuracy which is the best score amongst the chosen models. The accuracy score of LightGBM is improved by approximately 7% regarding the k-mer length of 3. Also, the RF is another model that exceeds 70% regarding accuracy. It can be seen that the model with the most performance increase is SVM considering accuracy which increased from 57% to 68%. It should also be noted that the AUC score of LightGBM is over the 80%. On the other hand, the Gaussian NB is the only model that decreased in every metric, especially in the kappa score. 

**Figure 9:**
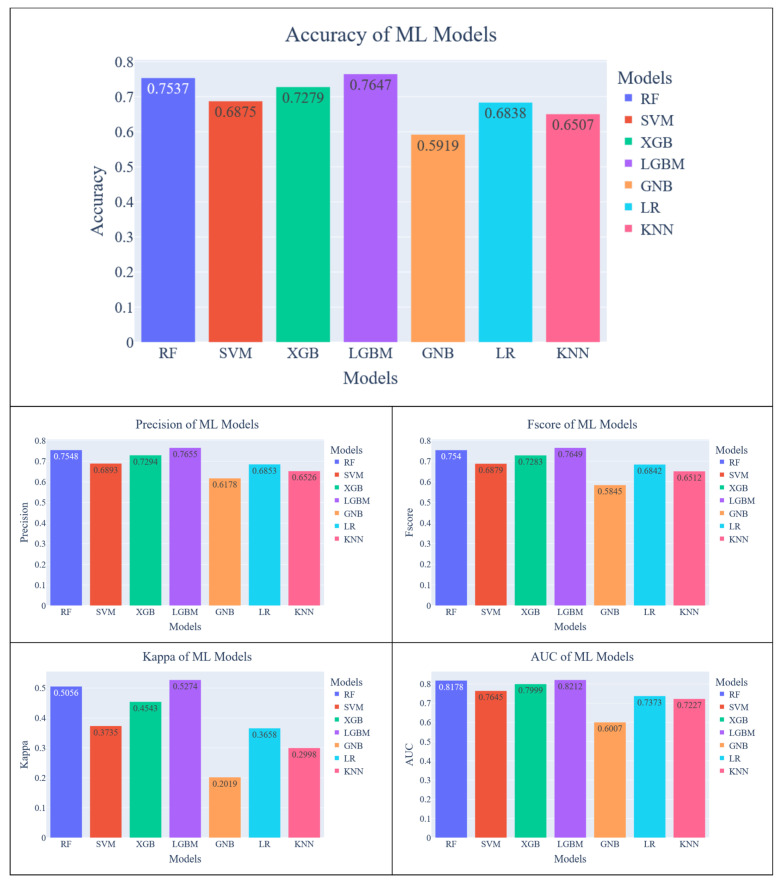
Evaluation results of ML models for k = 5.

In the third phase, the above-mentioned process is completed for a k-mer length of 5. The result can be seen in Figure 9. The LightGBM is the overachieving model regarding every metric, again. The accuracy of the LightGBM model exceeds 76% which is the best result among the all k-mer lengths. The RF model is also achieved over 75% accuracy. LightGBM and RF show similar results on every metric including kappa and AUC scores. Also, it can be seen that the XGB is the model whose performance has improved the most in terms of accuracy, which is above 72%. On the other hand, GaussianNB demonstrates the weakest performance on every metric including accuracy.

**Figure 10:**
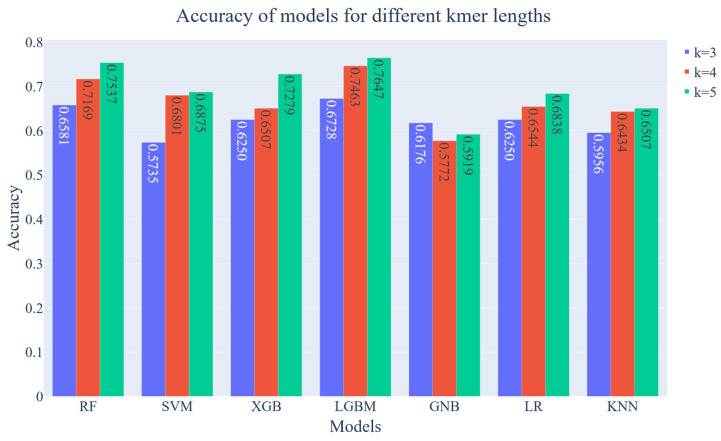
Accuracy of ML models on different k-mer lengths from 3 to 5.

To sum up, increasing the k-mer length enhanced the accuracy on all models except the GNB (Figure 10). While the LightGBM model outperformed other models for each k-mer length with over 76% accuracy, RF, also provided similar results. As the k-mer length was increased, the performance of XGBoost also showed a significant increase, reaching above 72% accuracy.

Here, it is worth mentioning and comparing the results with OTU-based methods because the majority of the IBD and other pathologies used OTU-based ML approaches. In the literature, Gevers et al.’s [1] dataset is used in different OTU-based studies. Asgari et al. [32] used 9511 OTU features to train RF and SVM to predict Crohn’s Disease. The study demonstrated 0.74 ± 0.04 and 0.68 ± 0.04 precision from RF and SVM models, respectively. In our study, the RF model presented 0.7548 precision for a k-mer length of 5 which is similar to the results of Asgari et al.’s study. 

Manandhar et al. [43] used two datasets including Gevers et al.’s dataset and the RF model to predict IBD. This study not only employs OTU-based features but also 50 differential bacterial taxa for this task. The RF model demonstrated 74 ± 2% for IBD prediction task using the OTU-based approach which is again similar to our results.

Linares-Blanco et al. [44] used only fecal samples to generate metagenomic signatures and tested ML models with two datasets. The RF model presented a 0.76 AUC score which is below our RF and LGBM AUC scores. 

In order to examine the results in more detail, Mc Nemar’s test is used. Mc Nemar’s test is a statistical test used to analyze paired categorical data which can also be adopted to evaluate binary classification results [45]. The test is commonly applied to determine whether there is a significant difference in the proportions of a particular attribute between two conditions. This difference is named as the *z* score and is calculated using Equation (1) below:

(1) $$z=\frac{\left(|N_{sf}-N_{fs}|-1\right)}{\sqrt{N_{sf}+N_{fs}}}$$

Here, $N_{sf}$ and $N_{fs}$ correspond to the number of paired observations where one ML model succeeds and the other one fails. If two ML models output the same prediction, the *z* score will be zero which means there is no significant difference between performance of the two models. Moreover, it is interpreted that the performance of the two models is statistically different as the *z* score increases. *z* scores and corresponding confidence levels for one-tailed and two-tailed predictions can be seen in Table 1. Here, one-tailed prediction value is high when one ML model is overperforming than other, and two-tailed prediction value shows the difference between two ML models.

In Table 2, Mc Nemar’s test results can be seen in which the ML model performed better in the provided datasets and is indicated by the arrowheads. The statistical significance results are indicated by the z scores that are provided next to the arrowheads. By examining this table, we can once again see the superior performance of LGBM which is represented by arrowheads in each comparison. LGBM demonstrates over 90% confidence compared to SVM, XGB, GNB, LR and KNN. The lowest z-score of LGBM is generated comparing RF which is 0.4. Furthermore, RF has also produced better results compared to all models except LGBM. This table is further proof that this study produces statistically significant results comparing these ML models on this dataset.

To further test the performance of the models a second dataset is employed. This dataset is obtained from Jacobs et al.’s study and includes samples from 90 individuals [46] (Bioproject PRJNA324147 [46]). These individuals are from 21 families with pediatric inflammatory bowel disease. The dataset contains 26 Crohn’s Disease patients, 10 Ulcerative Colitis patients, and 54 healthy siblings/parents. All of the data in the dataset were obtained from stool samples. As mentioned before, the main dataset contains both stool and biopsy samples. Moreover, the second dataset includes not only child samples, but also their parents’ while the main dataset only includes child patients.

**Figure 11:**
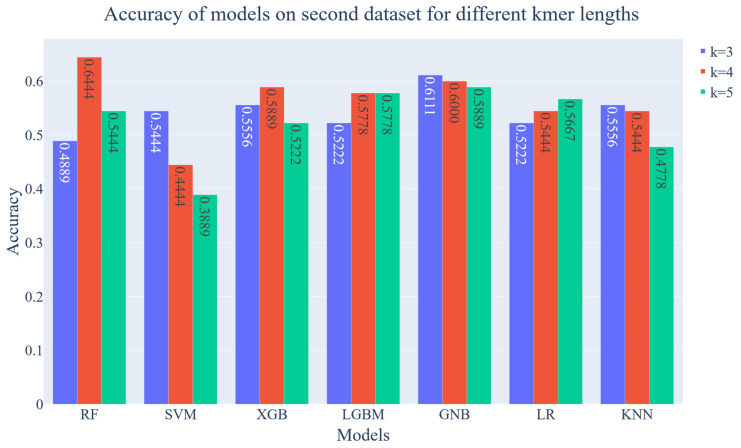
Accuracy of ML models on second dataset for k-mer lengths from 3 to 5.

The new dataset was used to test the previously trained and optimized ML models. It is important to note that the hyperparameters are optimized with the main dataset. The accuracy of the results can be seen in Figure 11. As can be seen in the figure, the best result was obtained as 64.44% accuracy using the RF algorithm with a k-mer length of 4. The GNB algorithm demonstrated the second best results with 61.11% accuracy. The results demonstrated that there is no correlation between k-mer length and accuracy for the second database. There could be several reasons for these results. First, the second dataset contains not only child patients but also adult patients. Also, two datasets consist of samples from different geographical regions which can cause diversity in the microbiota. Lastly, the dataset contains samples from only 21 family which can cause bias. For now, it is hard to find samples of the V4 region of the 16S rRNA gene for IBD. The literature does not have large datasets which are required for ML models to learn and to consistently distinguish between diseased and healthy individuals.

## 5. Conclusions

The use of microbiota data in ML has the potential to revolutionize our understanding of the human microbiome and its role in health and disease. By enabling more accurate and personalized care for patients, this approach could lead to improved health outcomes and a better understanding of the complex interplay between microbiota and human health. 

In this study, popular ML models were utilized to predict IBD from k-mer representations of 16S rRNA sequence data of patients. The dataset used in the study contains microbiota samples taken from 1359 individuals. When compared with other ML approaches mainly based on OTU/ASV features, k-mer based ML tools are alignment-free approaches which minimize the bioinformatics analysis steps including clustering and taxa assignment. Moreover, unknown taxa or annotation errors may limit performance of feature-based ML classification approaches. 

In the preprocessing stage, sequence identifiers and quality scores were removed from the fastq file so that only nucleotide sequences remain. Secondly, these raw sequences were converted in De Bruijn graph representation form with k-mer lengths of 3, 4 and 5. Later, these representations were converted into a structured form to train ML models. 

Seven ML models including Random Forest, Support Vector Machines, XGBoost, Light Gradient Boosting Machine, Gaussian Naïve Bayes, Logistic Regression and k-Nearest Neighbor were employed. The hyperparameter optimization was achieved using the Grid Search algorithm. The evaluation metrics of accuracy, precision, fscore, kappa and area under the receiver operating characteristic curve (AUC) were chosen. 

The ML models were trained and tested with three sets of data. The best model was LightGBM with 76.47% accuracy for a k-mer length of 5. Also, results were statistically significant regarding Mc Nemar’s test results. In future work, larger k-mer lengths will be chosen and Deep Learning models will be employed.

Analyzing microbiome data using ML techniques offers promising ways for enhancing the diagnosis and treatment of IBD. The human microbiome plays a crucial role in maintaining body health and changes in microbiome can be linked with various diseases. ML can help resolve complex relationships within microbiome data to help the diagnosis of IBD. By using different ML models forecasting disease progression, response to treatment based on microbiome data can be possible. ML algorithms require large datasets to accurately predict the diseases. By using next generation sequencing techniques, more and more sequence data will be uploaded to the open access databases, and using these large datasets can elevate the performance of ML models. 

## Figures and Tables

**Table 1:** Some z scores and corresponding confidence levels [46].

Z Score	One-Tailed Prediction	Two-Tailed Prediction
1.645	95%	90%
1.960	97.5%	95%
2.326	99%	98%
2.576	99.5%	99%

**Table 2:** Mc Nemar’s Test results for k-mer length of 5.

		SVM	XGB	LGBM	GNB	LR	KNN
	
RF		←1.95	←0.8571	**↑0.4**	←4.6368	←2.7449	←3.1386
SVM			↑1.0846	**↑2.1952**	←2.6064	0	←1.2247
XGB				**↑1.3568**	←3.6552	←1.375	←2.1442
LGBM					**←4.6705**	**←2.8062**	**←3.4641**
GNB						↑2.7	↑1.5811
LR							←0.9363

## Data Availability

Data are available in a publicly accessible repository. The data presented in this study are openly available in reference number [1].
